# Superhydrophilic Silica Coatings via a Sequential Dipping Process

**DOI:** 10.3390/molecules30081857

**Published:** 2025-04-21

**Authors:** Junbao Xie, Anqi Liang, Qin Lin, Nantian Chen, Abbas Ahmed, Xiaoyan Li, Rongkun Jian, Luyi Sun, Fuchuan Ding

**Affiliations:** 1 Fujian Key Laboratory of Polymer Science, College of Chemistry and Materials Science, Fujian Normal University, Fuzhou 350007, China; qsz20221455@student.fjnu.edu.cn (J.X.); qsx20220946@student.fjnu.edu.cn (A.L.); 15282511845@163.com (Q.L.); 18859815639@163.com (N.C.); jrkht1987@fjnu.edu.cn (R.J.); 2 Polymer Program, Institute of Materials Science and Department of Chemical & Biomolecular Engineering, University of Connecticut, Storrs, CT 06269, USA; abbas.ahmed@uconn.edu

**Keywords:** silica, sequential dipping process, superhydrophilicity, underwater superoleophobicity, oil–water separation, anti-fouling

## Abstract

A superhydrophilic silica coating was prepared using a sequential dipping process involving acid-catalyzed silica, base-catalyzed silica, and 3-(trihydroxysilyl)propanesulfonic acid. Acid-catalyzed and base-catalyzed silica particles with varying diameters were synthesized by hydrolyzing tetraethyl orthosilicate using HCl and NH_3_·H_2_O as catalysts, respectively. 3-(Trihydroxysilyl)propanesulfonic acid was obtained by oxidizing mercaptopropyl trimethoxysilane with hydrogen peroxide under acidic conditions. The resulting silica coating exhibited exceptional superhydrophilicity, with a water static contact angle of 5.0°, and demonstrated underwater superoleophobicity, with a hexadecane underwater contact angle exceeding 140°. Surfaces coated with the superhydrophilic silica coatings showed excellent performances in oil–water separation, anti-protein adsorption, and anti-fogging applications.

## 1. Introduction

Superhydrophilic surfaces are characterized by water contact angles (WCAs) lower than 5° within 0.5 s and the near-instantaneous spreading of condensed water droplets [1]. These surfaces exhibit exceptional wetting properties, which are governed by the chemical composition and topographic structure of the surface [2]. Superhydrophilic coatings have huge potential for applications in self-cleaning, water/oil separation, anti-fogging, and anti-fouling [3,4,5,6,7,8]. Typically, superhydrophilic surfaces are achieved by combining hydrophilic materials with carefully engineered rough topographies [9].

Various methods can be used to fabricate superhydrophilic surfaces, including vapor deposition [10], ultrasonic spray pyrolysis [11], layer-by-layer (LbL) assembly [12], the sol–gel method [13], etching [14], electrospinning [15], interfacial polymerization [16], and bottom-up assembly [17]. Surface coating offers a simple and effective way to modify surfaces for biocompatibility and hydrophilicity without chemically altering the substrate [18,19]. Dip coating [20,21,22,23], spray coating [24,25,26], doctoral blading [27], and spin coating [28] are common surface coating techniques used to create hydrophilic surfaces [29]. Dip coating, also known as immersion precipitation, involves immersing a substrate in a liquid coating dispersion, withdrawing it at a controlled rate, and then drying or curing the coating [30].

Hydrophilic surfaces are primarily achieved through the use of nanoparticles [31], nanoparticle/polyelectrolyte composites [32], and polymer/polyelectrolyte composites [33]. Titanium dioxide (TiO_2_) nanoparticles [34] and silica (SiO_2_) nanoparticles [35] are commonly used hydrophilic components due to their diverse morphology and hydrophilic properties. TiO_2_-based coatings, however, require activation via UV exposure to maintain their super-wetting properties, limiting their practical applications in environments with light [36]. In contrast, silica nanoparticles possess an inherent hydrophilicity due to the abundance of surface hydroxyl groups [37]. Additionally, their network structure offers excellent hydrolytic stability. Unlike polymer-based coatings, which are susceptible to swelling and cracking after prolonged exposure to aqueous environments, silica nanoparticles are widely employed in the fabrication of superhydrophilic surfaces. The formation of superhydrophilic properties also relies on microstructural roughness, which can be tailored using techniques such as LbL assembly [38], chemical vapor deposition (CVD) [39,40], and the sol–gel method [41]. Among these, the sol–gel method is usually favored due to its simpler process, lower costs, and eco-friendliness [42]. However, the superhydrophilic surfaces solely based on silica nanoparticles can be prone to fouling and a loss of oil repellency over time, limiting their long-term applicability [43].

In this work, acid- and base-catalyzed silica nanoparticles were synthesized by hydrolyzing tetraethyl orthosilicate (TEOS) using HCl and NH_3_·H_2_O as catalysts, respectively. Additionally, 3-(trihydroxysilyl)propanesulfonic acid was prepared by oxidizing mercaptopropyl trimethoxysilane with hydrogen peroxide under acidic conditions. A superhydrophilic coating, incorporating the above components, was fabricated via facile dip coating. The resulting coating exhibited a surface hardness of 2H, adhesion grade of 0, high abrasion resistance, superhydrophilicity, and underwater superoleophobicity. This coating shows great potential for applications including oil–water separation, anti-protein adsorption, and anti-fogging.

## 2. Results and Discussion

**Figure 1:**
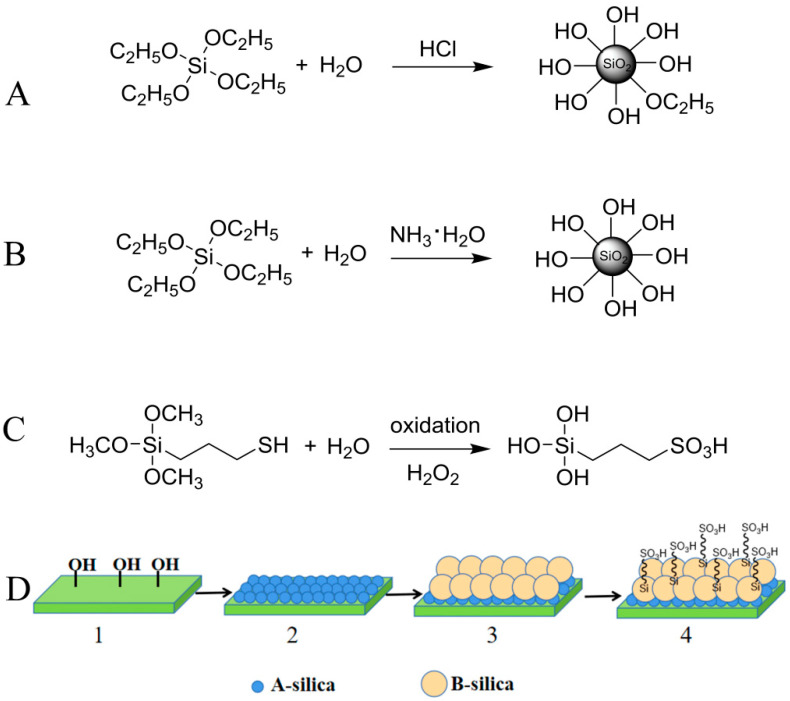
Schematic of synthesis process: (**A**) A-silica, (**B**) B-silica, and (**C**) TPS, and (**D**) preparation process and structure of formed ABS-silica coating.

As shown in Figure 1A,B, A-silica and B-silica were prepared with HCl and NH_3_·H_2_O as catalysts, respectively, following the Stöber method, [44,45]. During the oxidation of MPTMS using hydrogen peroxide in an acidic aqueous solution, the sulfhydryl group in MPTMS was oxidized to a sulfonic acid group and the methoxy groups were hydrolyzed to hydroxyl groups, forming TPS (Figure 1C).

The cleaned glass slides, PET films, nylon fabrics, and medical cotton were immersed sequentially in the A-silica, B (or B′, B″)-silica, and TPS dispersions. The schematic of the sequential coating process and structure of the formed ABS-silica coating are shown in Figure 1D.

**Figure 2:**
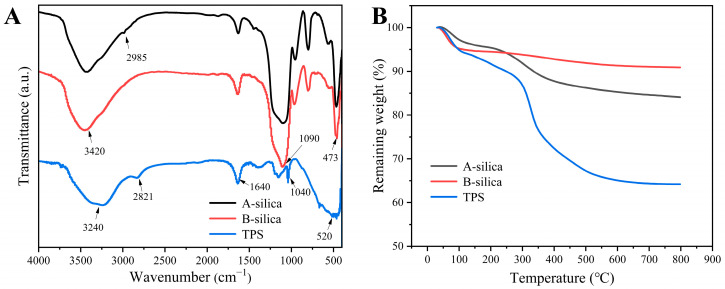
(**A**) FTIR spectra and (**B**) TGA thermograms of the A-silica, B-silica, and TPS.

The FTIR spectra of the samples are presented in Figure 2A. The absorption bands at 1090 and 473 cm^−1^ in the A-silica and B-silica correspond to the stretching and bending vibrations of the Si-O-Si bonds, respectively [46,47]. The broad absorption band at ~3420 cm^−1^ is attributed to the stretching vibrations of Si-OH groups [48]. Under acidic conditions, the TEOS was not fully hydrolyzed, leaving a small amount of ethoxy groups, which results in a characteristic C-H absorption peak at 2985 cm^−1^ [49]. In contrast, the TEOS underwent complete hydrolysis under basic conditions. The absorption band at 1640 cm^−1^ in the A-silica, B-silica, and TPS is assigned to the bending vibration of O-H groups from the adsorbed water. For TPS, the absorption bands at 1040 and 520 cm^−1^ are characteristic of the -SO_3_H group [50]. Additionally, the absorption peak at 2821 cm^−1^ in TPS arises from the stretching vibration of the C-H bonds in the –CH_2_CH_2_SO_3_H group [51].

Figure 2B presents the TGA thermograms of the A-silica, B-silica, and TPS. The thermograms of the A-silica and B-silica reveal two distinct mass loss stages. In the range of 30–180 °C, the weight loss is primarily attributed to the evaporation of adsorbed moisture, with the A-silica losing 4.4% of its mass and the B-silica losing 5.5%. The slightly higher moisture content of the B-silica is likely due to its more complete hydrolysis and higher surface hydroxyl group density compared to the A-silica. The second stage, from 200 to 650 °C, is characterized by weight loss due to dehydration condensation and the detachment of ethoxy groups on the silica surface. The A-silica exhibits a weight loss of approximately 11.3%, compared to 3.7% for the B-silica. This notable difference is primarily attributed to the following mechanisms: First, the ethoxy groups in A-silica are more susceptible to chemical bond cleavage. Additionally, they have a higher molecular weight than hydroxyl groups. Second, the smaller particle size of A-silica particles provides a larger specific surface area, increasing the density of surface hydroxyl groups (–OH). Third, the high content of surface hydroxyl groups releases more H_2_O molecules during the high-temperature dehydration stage, resulting in a more substantial mass loss [52].

In contrast, the TGA thermogram of the TPS demonstrates notable differences. Between 30 and 180 °C, TPS shows a weight loss of approximately 7.6%, attributed to the hydrophilic nature of the sulfonic acid groups, which promote greater moisture adsorption. From 200 to 650 °C, the weight loss is approximately 26.8%, primarily due to the decomposition of the sulfonic acid group, hydrocarbon segments, and the dehydration condensation [53,54].

**Figure 3:**
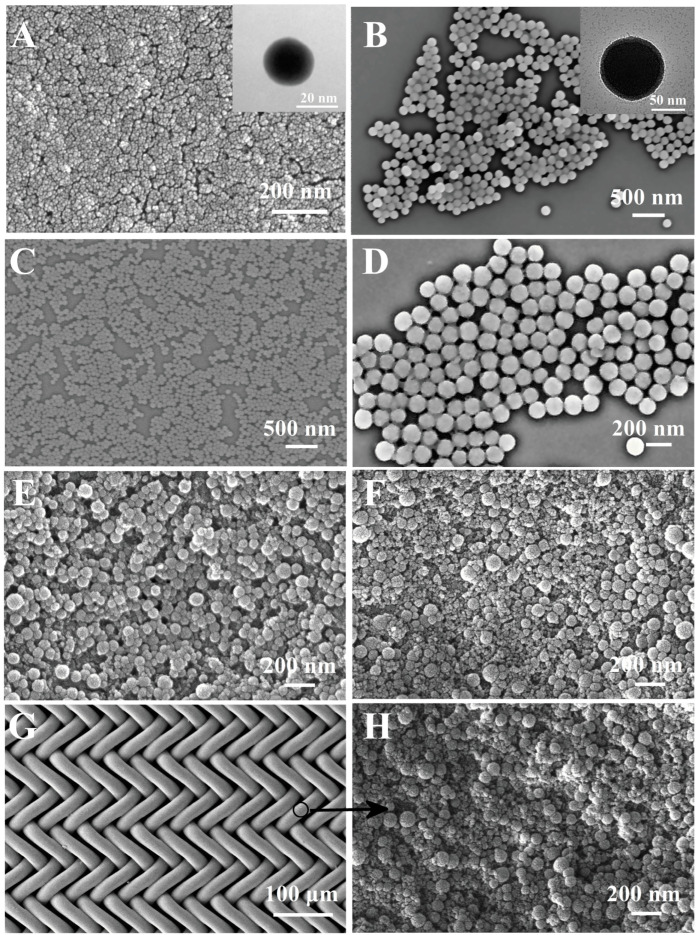
(**A**,**B**) SEM images of A-silica (**A**) and B-silica (**B**); insets in (**A**,**B**) show corresponding TEM images. (**C**,**D**) SEM images of B′-silica (**C**) and B″-silica (**D**); (**E**) SEM images of AB-silica coating on glass slide. (**F**) SEM image of ABS-silica coating on glass slide. (**G**) SEM image of coated nylon fabric. (**H**) SEM image of nylon fabric coated with ABS-silica coating.

The SEM images of the A-silica and B-silica are shown in Figure 3A and Figure 3B, respectively. The A-silica coating exhibits a typical nanoscale porous structure, composed of spherical silica nanoparticles with diameters of ca. 20 nm, while the B-silica particles are much larger, having a diameter of ca. 100 nm. Due to its smaller particle size and high hydroxyl group density, the A-silica was applied as the bottom layer to enhance the adhesion between the substrate and the B-silica, which will be discussed in more detail later. The SEM images of the B′-silica and B″-silica are shown in Figure 3C and Figure 3D, respectively. The diameter of the B′-silica particles is ca. 65 nm, while the diameter of the B″-silica particles is ca. 150 nm. This observation suggests that the NH_3_·H_2_O catalyst concentration significantly influences the particle size of the silica formed via base catalysis; a lower catalyst content results in a corresponding decrease in silica particle size, which is consistent with earlier reports [55,56,57]. After coating B-silica on top of the A-silica layer, the surface roughness of the formed AB-silica coating was enhanced due to the larger particle size of B-silica, as shown in Figure 3E. TPS, known for its strong hydrophilicity, was coated as the top layer to endow the coating with superhydrophilic properties. Figure 3F demonstrates that after applying TPS onto the B-silica layer, the formed ABS-silica coating retains its high surface roughness.

Figure 3G,H reveal that the nylon fabric, after being coated with the ABS-silica coating using the same sequential dip coating method, exhibits a surface morphology similar to that of the ABS-silica coating on cleaned glass slides. This result highlights the adaptability of the coating method to various substrates.

Using this sequential dip coating method, the first layer of the small A-silica particles enhances the adhesion to the substrate, the second layer of larger basic silica particles improves the surface roughness, and the third layer of TPS introduces polar hydrophilic groups, further increasing the substrate’s hydrophilicity [58]. Condensation reactions between each layer can improve the layer adhesion [59,60].

**Figure 4:**
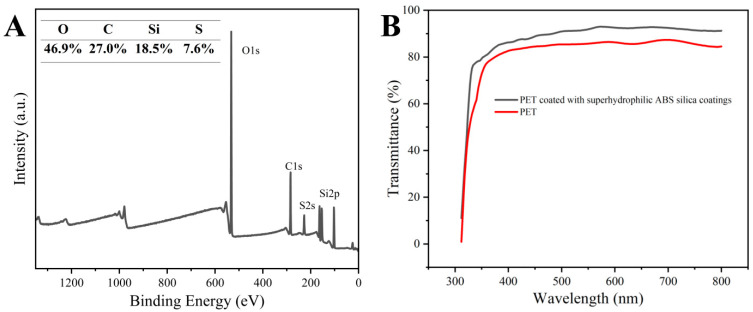
(**A**) XPS survey spectrum of ABS-silica coatings and (**B**) transmittance of uncoated PET and PET coated with ABS-silica coating.

The presence of sulfur in the outer layer of the ABS-silica coatings was confirmed by the XPS analysis, as shown in Figure 4A. The XPS peaks at 533.1, 291.9, 229.0, and 103.8 eV correspond to O 1s, C 1s, S 2s, and Si 2p, respectively [61]. These results confirm the successful grafting of TPS onto the coating surface. The presence of sulfonic acid groups in the TPS on the coating surface enhances the surface polarity, a critical factor for achieving superhydrophilicity [62].

The UV-Vis transmittance spectra of the uncoated PET and the PET coated with the ABS-silica coating are presented in Figure 4B. The coated PET film exhibited a slightly higher transmittance compared to the uncoated PET, which can be attributed to the antireflection effect [63]. This result indicates that the ABS-silica coating enhances the optical clarity of the substrate, making it suitable for applications where high optical transparency is essential.

The ABS-silica coating demonstrated a stronger adhesion than the BS-silica coating, as evaluated according to ISO standard 2409 [64] (Table 1), which rates coating adhesion on a scale from 0 to 5, with 0 indicating the highest adhesion. The ABS-silica and BS-silica coatings received adhesion grades of 0 and 3, respectively, confirming the firm attachment of the ABS-silica coating to the glass substrate. Similarly, the wear damage analysis based on ASTM standard D4060 demonstrated that the ABS-silica coating exhibited a greater wear durability than the BS-silica coating [65]. After 300 friction cycles, the ABS-silica coating retained its excellent superhydrophilic properties, further confirming its strong wear resistance, whereas the BS-silica coating sustained only 50 friction cycles before significant degradation. This enhanced adhesion and durability are attributed to the smaller particle size and higher hydroxyl group density of A-silica, which promote stronger bonding with the glass substrate. Additionally, the effective interlayer bonding further improves the overall mechanical performance of the ABS-silica coating [66].

A pencil hardness test was adopted to assess the hardness of the ABS-silica and BS-silica coatings, revealing a hardness level of 2H for both, as anticipated. The high hardness, along with the superior adhesion and durability of the ABS-silica coating, as discussed above, makes it highly advantageous for various surface-related applications.

**Figure 5:**
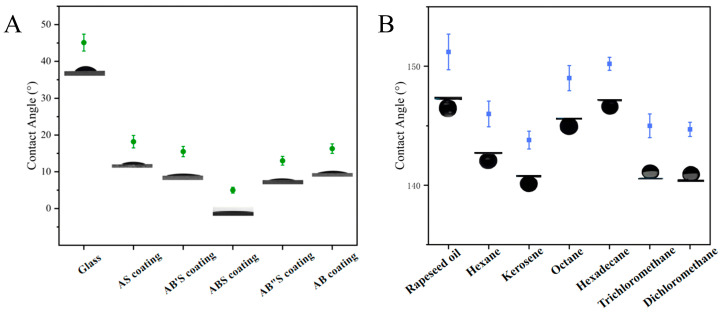
(**A**) Water contact angles of the uncoated and coated glass slides and (**B**) underwater oil contact angles of the ABS-silica coating.

The wettability of the silica coating was systematically evaluated by measuring the static water contact angle and underwater oil contact angle. As shown in Figure 5A, the water contact angle of an uncoated glass slide was measured at 45.1° ± 2.3°, indicating its moderate wettability. In contrast, the static water contact angles of the AS-, AB′S-, ABS-, and AB″S-silica coatings were 18.2° ± 1.7°, 15.5° ± 1.4°, 5.0° ± 0.8°, and 13.0° ± 1.2°, respectively. This observed trend can be attributed to variations in the particle size of the silica layers, which were controlled by adjusting the concentration of the base catalyst, ammonium hydroxide (NH_3_·H_2_O). Specifically, for AS-, AB′S-, ABS-, and AB″S-silica coatings, the top silica layer consisted of particles with diameters of approximately 20 (A-silica), 65, 100, and 150 nm, respectively.

The AS-silica coating was formed by depositing A-silica particles with a diameter of 20 nm, resulting in a relatively smooth surface (Figure 3A). Such a smooth surface is suboptimal for achieving superhydrophilicity because it lacks the sufficient surface roughness to enhance the capillary action and promote rapid water spreading [53], leading to a relatively high contact angle of 18.2°. As the deposited silica particle size increased, the surface roughness also increased, which, combined with the inherently hydrophilic nature of silica, enhanced wettability and lowered the contact angle. The ABS-silica coating, which incorporated B-silica particles of ca. 100 nm, exhibited the lowest water contact angle (5.0°), indicating superhydrophilicity. However, when the particle size increased further to 150 nm in the AB″S-silica coating, the contact angle increased to 13.0°. This trend reversal can be explained by the Cassie–Baxter wetting state, where an excessively large surface roughness can lead to air entrapment, reducing the effective water spreading and thus increasing the contact angle [67].

To further confirm the role of the top TPS layer, a control AB-silica coating (without the top TPS layer) was fabricated and tested. This sample exhibited a water contact angle of 16.3° ± 1.3° (Figure 5A), demonstrating that the introduction of the TPS top layer is essential for achieving ultra-low contact angles. The TPS layer enhances hydrophilicity by providing additional polar functional groups that facilitate water spreading, further reducing the contact angle.

Figure 5B illustrates the underwater oil contact angles of the ABS-silica coating. Various oils, including n-hexane, n-octane, hexadecane, kerosene, rapeseed oil, dichloromethane, and trichloromethane, were tested, all yielding underwater oil contact angles exceeding 140.0°. These findings demonstrate that the ABS-silica coating possesses excellent hydrophilic and underwater oleophobic properties, achieving the desired superhydrophilic and underwater oleophobic performance.

Given that the ABS-silica coating demonstrates an optimal superhydrophilic and underwater oleophobic performance, along with superior durability, adhesion, and hardness, the following application demonstrations, including oil–water separation, anti-protein adhesion, and anti-fouling, were conducted exclusively on the ABS-silica coating.

**Figure 6:**
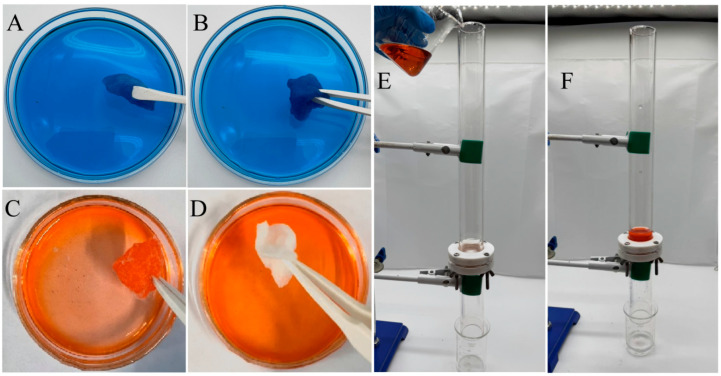
(**A**,**B**) Untreated (**A**) and ABS-silica-coated (**B**) medical cotton soaked in aqueous solution; (**C**,**D**) untreated (**C**) and ABS-silica-coated (**D**) medical cotton soaked in oil solution; and (**E**,**F**) oil/water separation demonstration using nylon fabric treated with ABS-silica coating.

Figure 6A,B illustrate the soaking behavior of the untreated and ABS-silica-coated medical cotton in water, while Figure 6C,D depict their soaking behavior in an oil (n-hexadecane). These images highlight the water absorption and oil-repelling properties of the medical cotton following the ABS-silica coating treatment. Notably, Figure 6B shows the coated cotton immersed in a methylene blue-stained aqueous solution. The cotton absorbs water readily, becoming wet and stained with the blue dye, suggesting its strong water absorption capability. In Figure 6C,D, the untreated and ABS-silica-coated medical cotton were immersed in n-hexadecane dyed with Sudan III for 10 s, respectively. The untreated cotton became visibly wet with oil and stained by the dye, whereas the coated cotton remained clean and free from contamination after its removal from the solution. These tests clearly demonstrate that the superhydrophilic ABS-silica coating imparts cotton with remarkable superhydrophilic and superoleophobic properties, effectively repelling oil.

The oil/water separation performance testing and demonstration are presented in Figure 6E,F, using the nylon fabric treated with the superhydrophilic ABS-silica coating. As shown in Figure 6E, the oil/water mixture consisted of 20 mL of deionized water and 20 mL of n-hexane dyed red with Sudan III (Macklin Biochemical Co., Ltd., Shanghai, China). To achieve uniform mixing, the oil and water were mixed using ultrasonic vibration. During the test, the treated nylon fabric was securely clamped between two glass tubes. The oil/water mixture (1:1 by volume) was charged into the upper glass tube. Due to the superhydrophilic properties of the filter cloth, nearly all the water (20 mL) passed rapidly through the coated filter solely under gravity and was collected in the lower beaker. Simultaneously, almost all the n-hexane (20 mL) was retained above the coated filter. The water collected in the lower beaker was clear and colorless, with no trace of red n-hexane, indicating effective separation.

This remarkable performance is attributed to the superhydrophilicity and underwater superoleophobicity of the treated nylon filter. These properties create a hydration layer on the substrate surface, preventing n-hexane droplets from wetting or penetrating the filter. The results visually demonstrate the excellent oil/water separation capabilities of the nylon fabric treated with the superhydrophilic ABS-silica coating.

**Figure 7:**
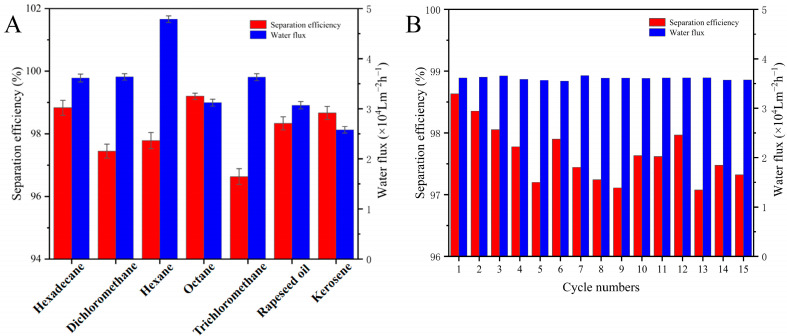
(**A**) Separation efficiency of nylon fabric coated with superhydrophilic ABS-silica for various oil–water mixtures. (**B**) Separation efficiency of nylon fabric coated with superhydrophilic ABS-silica for hexadecane–water mixture during cycling experiments.

The separation efficiency, water flux, recyclability, and durability of the coated nylon fabric were systematically evaluated, with the results shown in Figure 7. As seen in Figure 7A, the nylon fabric treated with the superhydrophilic ABS-coating exhibited a separation efficiency exceeding 96.0% for various oil/water mixtures (vol/vol = 1:1), including hexadecane, dichloromethane, n-hexane, n-octane, trichloromethane, rapeseed oil, and kerosene, demonstrating an excellent separation performance and indicating its broad applicability in oil/water separation. The water flux for n-hexane reached 48,000 L·m^−2^·h^−1^.

To test the recyclability and durability of the coated nylon fabric, multiple cycles of separation experiments were conducted, as shown in Figure 7B, using a hexadecane/water mixture as the sample. During the first separation of the hexadecane/water mixture, the treated nylon fabric achieved a separation efficiency of approximately 98.8%. After 15 cycles, the oil/water separation efficiency remained above 96.0%. These results demonstrate that the superhydrophilic ABS-silica coated nylon fabric not only provides an excellent oil/water separation efficiency, exceeding 96.0%, but is also recyclable, significantly enhancing its practicality.

**Figure 8:**
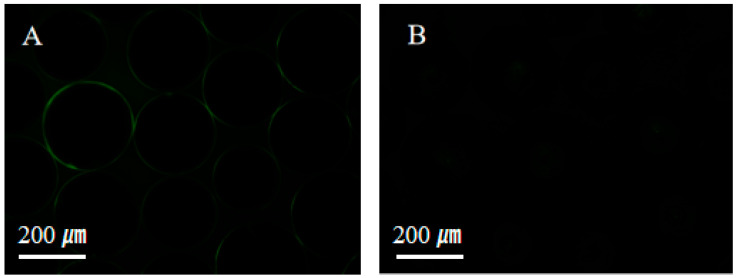
Fluorescence microscopy images of (**A**) uncoated glass beads and (**B**) glass beads coated with the superhydrophilic ABS-silica coating.

The resistance to protein adsorption was evaluated to assess the anti-fouling properties of the superhydrophilic ABS-silica coating, as shown in Figure 8. Bovine serum albumin labeled with fluorescein isothiocyanate (BSA-FITC) was used as a model biofoulant [68]. Uncoated glass beads readily adsorbed BSA-FITC, exhibiting strong fluorescence signals (Figure 8A). In contrast, glass beads coated with the superhydrophilic ABS-silica coatings showed a significantly reduced protein adsorption, with minimal fluorescence observed. This result demonstrates that the superhydrophilic ABS-silica coating effectively resists protein adsorption, highlighting its anti-fouling capability.

**Figure 9:**
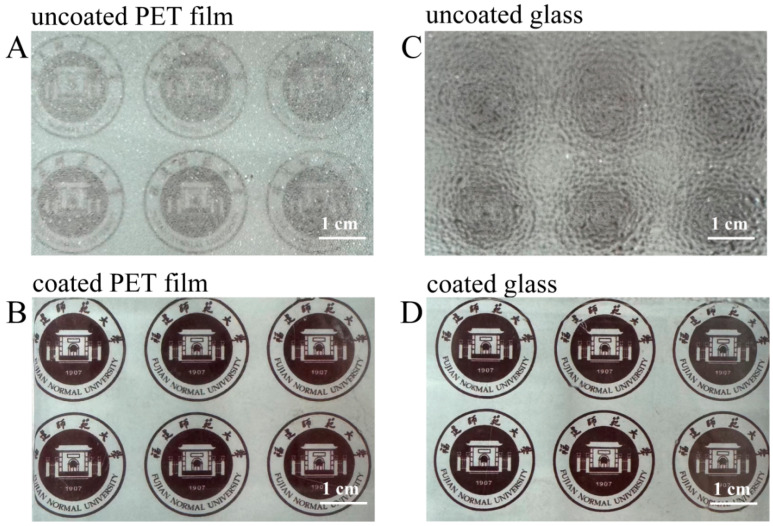
Photos from anti-fogging tests: (**A**,**B**) PET film substrate and (**C**,**D**) glass slide substrate.

The anti-fogging properties of the superhydrophilic ABS-silica coatings were evaluated using the PET film and glass slide as substrates. The uncoated and coated substrates were placed above hot water, and their light transparency was assessed after an exposure to water vapor for 10 s (Figure 9). On the uncoated PET film and glass surfaces, a fog layer formed, rendering the surfaces opaque. In contrast, the coated PET film and glass slides maintained high transparency under the same conditions. This outcome can be attributed to the superior hydrophilic nature of the superhydrophilic ABS-silica coating, which prevents fog formation by promoting a uniform water film dispersion [69].

## 3. Materials and Methods

### 3.1. Materials

Tetraethyl orthosilicate (TEOS, 99%) and 3-mercaptopropyl trimethoxy silane (MPTMS, 99%) were purchased from Sigma-Aldrich (St. Louis, MO, USA). Hydrogen peroxide (H_2_O_2_, 30%), nitric acid (HNO_3_), ethanol (99.5%), ammonium hydroxide (NH_3_·H_2_O, 25 wt. % in water), and dichloromethane (CH_2_Cl_2_) were sourced from Sinopharm Chemical Reagent Co., Ltd. (Shanghai, China). The nylon fabric, supplied by Hengyi Petrochemical Co., Ltd. (Hangzhou, China), has a thickness of 0.4 mm. Medical cotton was sourced from Johnson & Johnson (New Brunswick, NJ, USA). Microscope slides (75 mm × 25 mm) were supplied by VWR International, LLC (Radnor, PA, USA). Polyethylene terephthalate (PET) films with a thickness of 22 μm were obtained from Toyobo Plastics Co., Ltd. (Osaka, Japan). Fluorescein isothiocyanate (FITC)-labeled bovine serum albumin (BSA) (BSA-FITC) was supplied by Arbio Technology Co., Ltd. (Beijing, China).

### 3.2. Preparation of Superhydrophilic Silica Coating via Sequential Dipping

Acid-catalyzed silica (A-silica) was synthesized using HCl as the catalyst through a sol–gel process following the Stöber method [70]. Specifically, 7.20 g of deionized water, 20.00 g of TEOS, 200.00 g of absolute ethanol, and 10.00 g of HCl (1.00 wt. %) were mixed and stirred for 10 min. The solution was then kept stirring at 65 °C for 4 h to prepare A-silica. Base-catalyzed silica was synthesized using NH_3_·H_2_O as the catalyst through a sol–gel process following the Stöber method [47,71,72]. Specifically, to prepare base-catalyzed silica with different particle sizes, 1.00 g of deionized water, 1.00 g of TEOS, and 10.00 g of anhydrous ethanol were mixed with 0.50, 1.00, and 2.00 g of ammonium hydroxide solution, respectively, and stirred at 45 °C for 4 h to obtain B′-silica, B-silica, and B″-silica, respectively. 3-(Trihydroxysilyl)propanesulfonic acid (TPS) was prepared by oxidizing MPTMS with hydrogen peroxide under acidic conditions. In this process, 1.00 g of MPTMS was mixed with 3.00 g of deionized water and ultrasonicated for 20 min. Next, 6.00 g of hydrogen peroxide solution (H_2_O_2_, 30%) was slowly added to the MPTMS/water mixture at room temperature under mechanical stirring [73]. Hydrochloric acid was then used to adjust the pH of the solution to 6.0, and the mixture was stirred continuously for 24 h to complete the oxidation, resulting in a transparent TPS solution. The resulting A-silica, B-silica (and B′-silica and B″-silica), and TPS were diluted to 0.5 wt. % in ethanol for coating.

Prior to coating, the glass slides were treated with piranha solution (concentrated sulfuric acid/hydrogen peroxide = 7:3) for 30 s, followed by thorough rinsing with deionized water (2–3 times) and natural drying [74]. This treatment introduces hydroxyl groups on the surface, ensuring effective modification. The cleaned glass slides, PET films, nylon fabrics, and medical cotton were sequentially immersed in A-silica, B-silica (or B′-silica, B″-silica), and TPS dispersions for 5 s each. After immersion, the substrates were withdrawn at a rate of ca. 80 mm/min and dried at 80 °C for 2 min after each coating cycle. The first layer of coating, consisting of A-silica, was designated as the A-silica coating. After drying the A-silica coating, a second layer of B-silica was applied, resulting in the AB silica coating (i.e., A-silica + B-silica). Finally, after the AB-silica coating was dried, a third layer of TPS was applied, yielding the ABS-silica coating (i.e., A-silica + B-silica + TPS).

Moreover, a series of control samples were prepared for comparison purposes, including AB-silica coating (i.e., A-silica + B-silica), AS-silica (i.e., A-silica + TPS), BS-silica coating (i.e., B-silica + TPS), AB′S silica coating (i.e., A-silica + B′-silica + TPS), and AB″S silica coating (i.e., A-silica + B″-silica + TPS).

### 3.3. Characterization

Contact angles (CAs) were measured using a drop shape analyzer (DSA-25, Kruss Scientific Instrument Co., Hamburg, Germany) at room temperature, with 5 μL droplets of deionized water or hexadecane placed on the coating surface [74,75]. For water contact angle measurements in air, the water droplet was directly applied to the substrate. For underwater oil wetting performance characterization, the substrates were immersed in a transparent quartz container filled with water [76]. The CAs were determined by averaging ten measurements from ten different locations on the coating surface.

Fourier-transform infrared (FTIR) spectroscopy was performed using a Nicolet 577 spectrometer (Thermo Fisher Scientific, Waltham, MA, USA) with the KBr pellet method. The A-silica, B-silica, and TPS were ground separately, mixed with KBr, and pressed into transparent pellets.

The surface morphologies of the coatings were characterized by field emission scanning electron microscopy (FE-SEM, JEOL 7800F, Tokyo, Japan). Transmission electron microscopy (TEM, JEOL JEM-2100F, Tokyo, Japan) was used to observe the morphology of individual silica nanoparticles.

The A-silica and B-silica samples were freeze-dried and then placed in a vacuum oven at 60 °C to dry. The samples were uniformly spread as a thin layer at the bottom of a crucible. The thermogravimetric analysis (TGA) was conducted at a heating rate of 10 °C/min under a nitrogen atmosphere with a flow rate of 50 mL/min using a Q600 thermal analyzer (TA Instruments, New Castle, DE, USA).

Elemental analysis was performed using X-ray photoelectron spectroscopy (XPS) to analyze the chemical compositions of the ABS-silica coating.

The light transmittance of the PET film coated with the ABS-silica coating was measured using a UV-1750 ultraviolet-visible spectrophotometer (Shimadzu Corporation, Kyoto, Japan), with the wavelength range set from 300 to 800 nm.

Film hardness was measured according to ASTM D3363 standard using pencil hardness testing to assess coating durability [77]. Adhesion was evaluated using the ISO 2409 standard with the tape adhesion test, where a defined grid was cut into the coating, and a 3M 810 tape was applied and peeled off at a 60° angle [64]. Wear resistance was determined using ASTM standard D4060-10, employing a CS-10 abrader at a friction speed of 60 rpm and a 100 g weight load to assess the abrasion resistance of the ABS-silica coating [65].

### 3.4. Oil–Water Separation Test

The oil–water separation experiment was conducted using an oil–water separation device composed of two transparent glass tubes (diameter: 3.15 cm), with a nylon fabric coated with the ABS-silica coating clamped between them. First, the coated nylon fabric was immersed in deionized water to ensure it was thoroughly wetted. The wetted nylon fabric was then secured between the two glass tubes. Finally, the oil–water separation glass tubes were fixed onto a ring stand to conduct the oil–water separation experiment. A mixture of oil and water, with Sudan III dye added to the oil for visual tracking, was placed at the top of the test tube. The separation of oil and water occurred under the influence of gravity, allowing the process to be visually observed.

The efficiency of oil–water separation was calculated using the following formula:

(1) $$\eta =\frac{m_{1}}{m_{0}}\times 100\%$$

where m_0_ is the mass of the initial water, and m_1_ is the mass of the water collected after oil/water separation.

### 3.5. Anti-Protein Adsorption Test

The anti-protein adsorption performance of the ABS-silica coating was evaluated using a fluorescence inverted microscope (ECLIPSE Ts2R-FL, Nikon Corporation, Tokyo, Japan). Fluorescein isothiocyanate (FITC)-labeled bovine serum albumin (BSA-FITC) was employed as the detection substance to assess the protein anti-adsorption characteristics. The coated glass beads were immersed in a 0.10 mg/mL solution of BSA-FITC and incubated in the dark at 25 °C for 30 min. After incubation, the beads were washed three times with PBS solution to remove any unbound excess protein. The fluorescence intensity of the adsorbed fluorescent protein on the glass bead surfaces was then observed using the inverted fluorescence microscope, with a 385 nm light as the excitation source, to assess the anti-protein performance of the coating [78,79].

### 3.6. Anti-Fogging Test

The glass slides and PET films, with one half coated with the ABS-silica coating and the other half uncoated, were placed above boiling water (100 °C) to observe the anti-fogging effect on both the coated and uncoated sides. By comparing the fogging intensity under the same conditions, the performance of the ABS-silica coating in preventing fog was evaluated.

## 4. Conclusions

Silica particles of varying sizes were synthesized using hydrochloric acid and ammonium hydroxide as hydrolysis catalysts. A superhydrophilic ABS-silica coating with a rough nanostructured surface was then fabricated by sequentially depositing A-silica, B-silica, and TPS onto the substrate. This coating exhibited an excellent adhesion, wear durability, and hardness. The ABS-silica coating achieved a remarkably low static water contact angle of just 5°, while its underwater oil contact angle exceeded 140.0° for all tested oils, confirming its superior hydrophilicity and underwater oleophobicity.

When applied to the nylon fabric, the superhydrophilic ABS-silica coating demonstrated an exceptional oil–water separation efficiency, exceeding 96%. Furthermore, it maintained this high efficiency even after 15 separation cycles, highlighting its durability and recyclability. In addition to the oil–water separation, the ABS-silica coating exhibited a strong resistance to protein adsorption and excellent anti-fogging properties. Overall, the ABS-silica coating holds great promise for applications in oil–water separation, anti-fouling, and anti-fogging technologies.

## Figures and Tables

**Table 1:** Properties of BS and ABS-silica coatings on glass slides.

Test Item	BS-Silica Coating	ABS-Silica Coating
Adhesion grade	3	0
Pencil hardness	2H	2H
Wear cycles	50	300

## Data Availability

The original contributions presented in this study are included in the article. Further inquiries can be directed to the corresponding authors.
